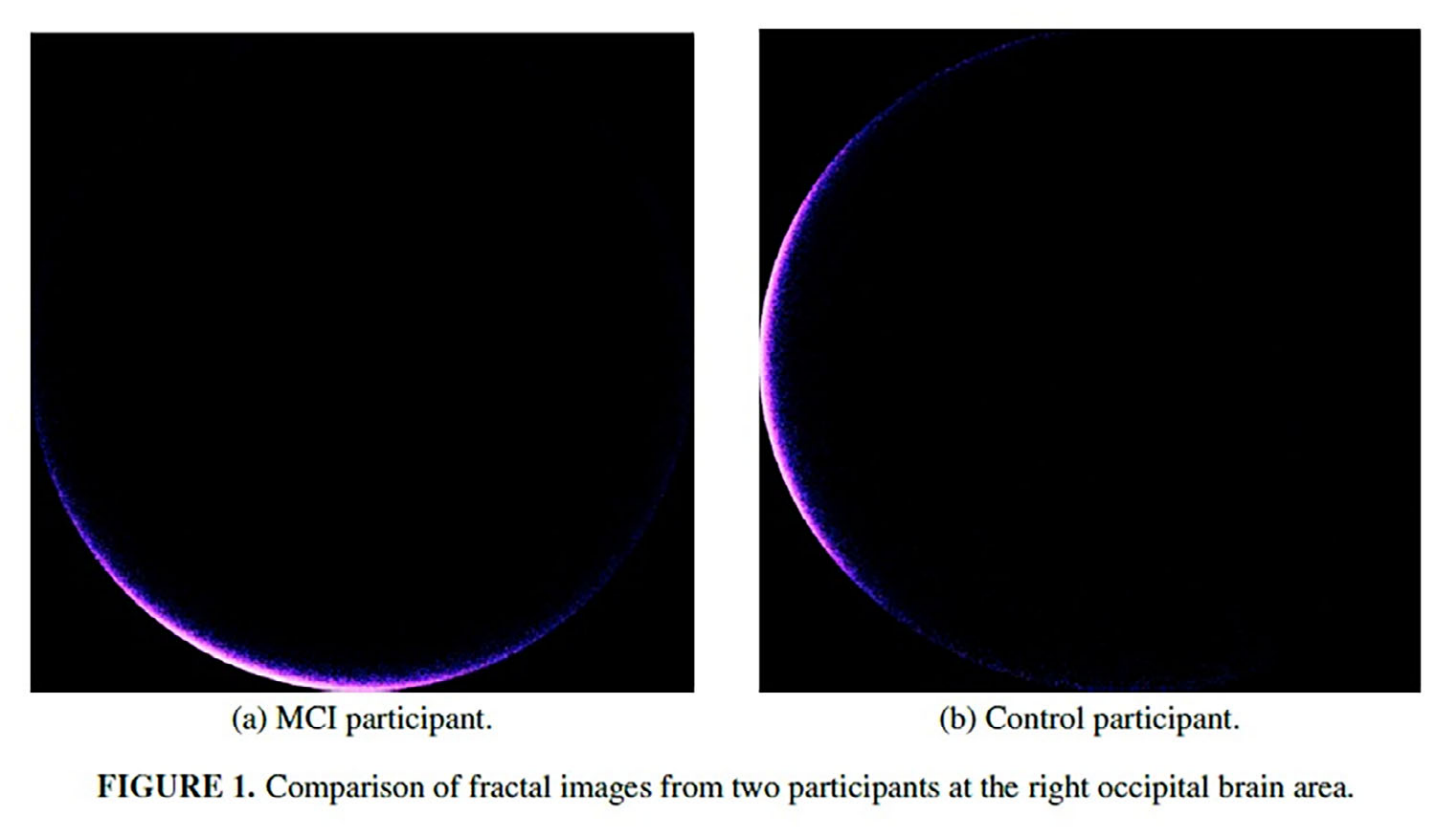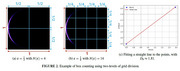# Resting‐State EEG in Mild Cognitive Impairment considering Fractal Dimensions and AI classifiers: a pilot study

**DOI:** 10.1002/alz70856_104968

**Published:** 2026-01-07

**Authors:** Brenda Fernanda Noguez‐Ruiz, Erika E. Rodríguez‐Torres, Felipe Humberto Contreras‐Alcalá, Eleni Mitsoura, Alejandra Rosales‐Lagarde

**Affiliations:** ^1^ Universidad Autónoma del Estado de México, Toluca, EM, Mexico; ^2^ Academic Area of ​​Mathematics and Physics, Autonomous University of the State of Hidalgo, Mineral de la Reforma, HG, Mexico; ^3^ Universidad Autónoma de la Ciudad de México, Mexico City, DF, Mexico; ^4^ Secretaría de Ciencia, Humanidades, Tecnología e Innovación, Mexico City, DF, Mexico; ^5^ Instituto Nacional de Psiquiatría Ramón de la Fuente Muñiz, Mexico City, DF, Mexico; ^6^ Universidad Latina, Mexico City, DF, Mexico

## Abstract

**Background:**

Mild Cognitive Impairment (MCI) is a neuropsychiatric syndrome with an alarming annual increase in Mexico. The Encuesta Nacional sobre Salud y Envejecimiento en México (ENASEM, Spanish for the National Survey of Health and Aging in Mexico) reports an estimated global incidence rate of 31.4 cases per 1,000 person‐years in subjects with non‐dementia cognitive impairment. In Mexico there is still a lack of systematic biological research. To address this gap, this study applied fractal techniques to analyze resting‐state EEG data.

**Method:**

Fractal analysis was performed on 5‐minute EEG recordings segmented into 1‐minute intervals from two groups of seniors at rest with their eyes closed (18 senior adults (10 control and 8 with MCI)): 48 recordings were used from the control group and 40 registers from the MCI group. Each of the free artifact minutes of the resting EEG were considered to obtain the fractal dimensions using the box‐counting method on circular‐shaped attractors generated through Barnsley's Chaos Game. These dimensions were then trained and analyzed to differentiate between groups using artificial intelligence (AI) classifiers. Four AI classifiers were selected for data training Extreme Gradient Boosting (XGBoost), Decision Tree, Support Vector Machines (SVM), and K‐Nearest Neighbors (KNN). Each classifier was thoroughly evaluated to determine the best fit for the dataset.

**Result:**

The results showed that the KNN classifier with three nearest neighbors and the Manhattan metric achieved the highest effectiveness. Additionally, a Student's t‐test was applied to compare the means of the fractal dimensions of each brain area. This analysis revealed a significant dissociation between the anterior and posterior brain areas. MCI appears to modify fractal dimensions of the bilateral frontal, right central and posterior areas.

**Conclusion:**

Therefore, fractal dimensions with the use of AI classifiers and costume statistics seem to offer a promising approach to the detection and physiological description of MCI.